# An uncommon presentation of hepatic hydatid cyst leading to biliary cirrhosis and portal hypertension 

**Published:** 2020

**Authors:** Hamid Asadzadeh-Aghdaei, Mohammad Reza Moshari, Reza Zandi, Mohammad Ali Karimi, Sina Salari, Pardis Ketabi Moghadam

**Affiliations:** 1 *Gastroenterology and Liver Diseases Research Center, Research Institute for Gastroenterology and Liver Diseases, Shahid Beheshti University of Medical Sciences, Tehran, Iran.*; 2 *Taleghani Hospital, Shahid Beheshti University of Medical Sciences, Tehran, Iran *

**Keywords:** Hepatic, Hydatid cyst, Biliary cirrhosis

## Abstract

Hydatid disease is an ongoing issue in endemic areas. Hydatid cysts can be seen in any organ but, liver is one of the most common involved organs. Cystobiliary communication as an overwhelming complication of hepatic hydatid cysts can contribute to the obstructive jaundice, cholangitis, sepsis and even biliary cirrhosis if left untreated. The patient we are trying to present is a 61-year-old farmer who presented with obstructive jaundice, multiple common bile duct stones and biliary cirrhosis attributed to a long-lasting untreated hepatic hydatid cyst. Portal hypertension is introduced to be an uncommon presentation of hydatid cyst. Extrinsic compression of the porta hepatis and obstruction of inferior vena cava are amongst major causes of hydatidosis leading up to portal hypertension as reported in the literature. Portal hypertension in the presented case is proposed to emerge from long-lasting cystobiliary communication ending in biliary cirrhosis.

## Introduction

 Cystic echinococcosis or hydatid disease is an ongoing issue in endemic areas. It is mainly caused by Echinococcus granulosus. Hydatid cysts can be seen in any organ but, liver is one of the most common involved organs. Although, this disease has been reported all over around the world, rural areas are more prone to that (1). Ultrasound has emerged as a paramount diagnostic modality assessing viability of the cyst and programming treatment (2). Cyst contents can be emptied into the biliary system, and leads to longstanding cystobiliary communication and eventually biliary cirrhosis (3).

## Case Report 

A 61-year-old man with past medical history of chronic obstructive pulmonary disease for 20 years and hepatic hydatid cyst surgery for 4 years ago presented to the emergency department of Taleghani hospital; a teaching referral hospital in Tehran, Iran, with recurrent abdominal pain aggravated by ingestion, obstructive jaundice, lower limbs edema and abdominal distention. On admission, he denied systemic symptoms like fever, chills and malaise. His vital signs were as follows: blood pressure 120/75 mmhg, heart rate 85 beats per minute, respiratory rate 16 per minute and O2 saturation 95% breathing room air. Before admission, his clinical scenario suggestive of biliary involvement made physicians proceed with an outpatient abdominopelvic ultrasound which revealed heterogeneous parenchymal echo pattern of liver with a large amount of ascites indicating the presence of concurrent cirrhosis and a heterogeneous 5.2x4.5cm cystic lesion with sharply defined but irregular borders in liver segment VIII. Common bile duct was reported dilated in proximal part and some sludge was detected in gallbladder. Serum aminotransferases demonstrated an elevation of about 3 times the upper limit of normal. Blood tests revealed a remarkable increase in ALP level up to 5 times the upper limit of normal. A total bilirubin level of 8.9 mg/dl was reported of which direct bilirubin 6.1 mg/dl was. Serum CA19-9 level was normal. Further evaluations by abdominopelvic CT scan were in line with ultrasonographic study. A CT scan revealed moderate ascites, increased gastrointestinal wall thickness, moderate generalized dilation of intra hepatic ducts, common bile duct and common hepatic duct with heterogeneous intraluminal filling defects and a 5.4x4cm-cystic lesion in right liver lobe adjacent to the scar of previous surgery with few calcified foci in its wall. An enlarged gallbladder with a dense layer in the neck suggestive of biliary sludge was also seen. Hydatid cyst treatment by albendazole 400 mg/BD started and he was transferred to the referral hospital for further evaluation and management of choledocholithiasis and cirrhosis. At the first day of admission, he was clearly jaundiced and his vital signs were stable. Ascites analysis revealed a high SAAG and low protein fluid with negative cytology for malignancy suggestive of cirrhosis necessitating a secondary work up.

**Figure-1:**
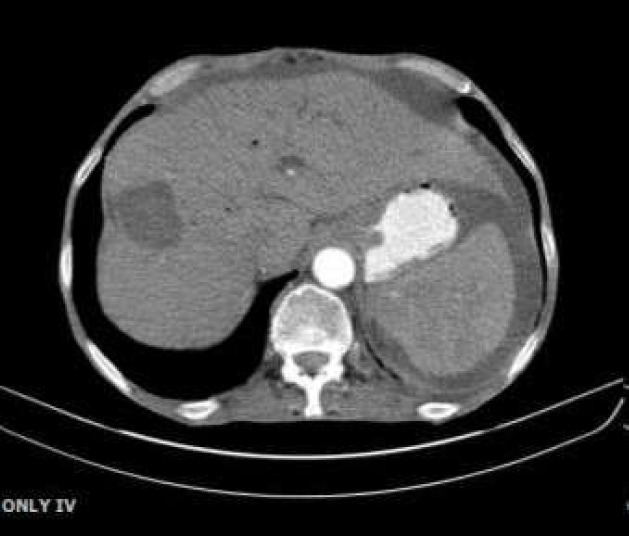
A 5.4x4cm-cystic lesion in right liver lobe

**Figure 2:**
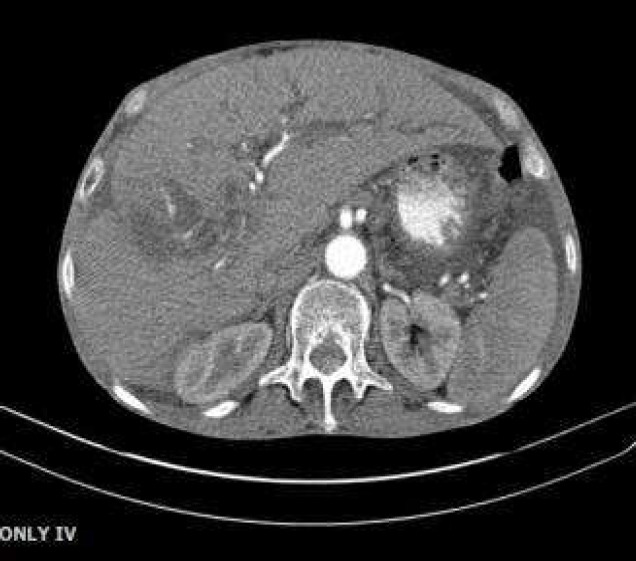
A5.4x4cm-cystic lesion in right liver lobe

**Figure-3:**
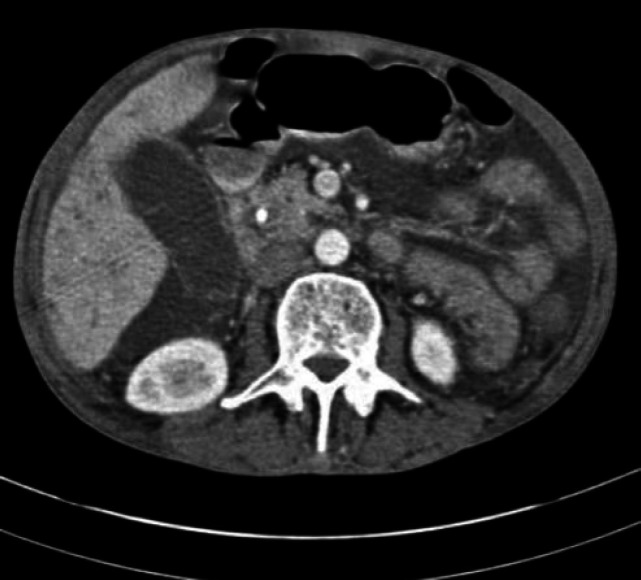
This picture shows a dilated CBD containing stent and heterogeneous filling defects compatible with multiple stones and hydatid cyst membranes

Given biliary obstruction, the patient underwent an ERCP. Duodenoscopy was then performed Common bile duct was measured 20mm with multiple large filling defects. We then performed dilation with balloon TTS 12-15 and more than 20 pigmented stones, sludge and pus were extracted by balloon. Finally, a 7cm, 10Fr-plastic stent was inserted. Another abdominopelvic CT scan was demanded as presented in figures 1-3.

**Figure 4:**
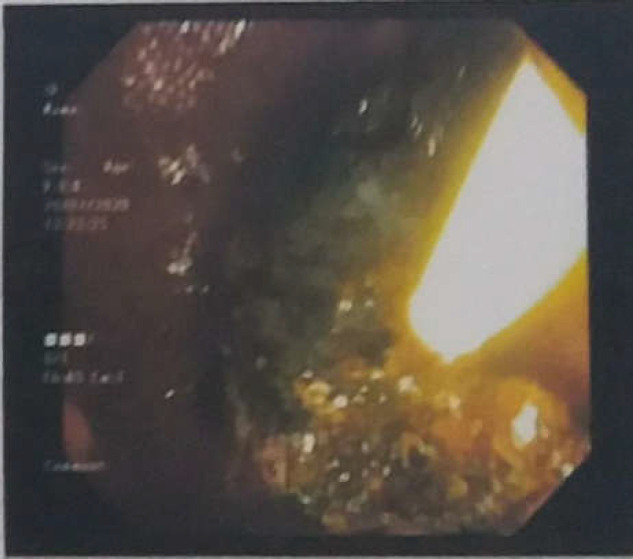
Extracted stones during ERCP

In spite of biliary stent placement, he continued to remain icterus. We decided to proceed with another ERCP. The procedure revealed more than 30 pigmented stones and hydatid cysts membranes in common bile duct which were extracted completely. An extracted stone has been disclosed in figure-4. Given the patient’s clinical picture, laboratory results, imaging and ERCP findings in addition to negative secondary work -ups for cirrhosis, we suspected a diagnosis of hydatid cyst with cystobiliary fistula formation resulting in a portal hypertension and cirrhosis. Portal hypertension is said to be one of the rare complications of hydatid cyst. It has been reported in cases afflicted by cysts involving liver hilum making a compression on porta hepatis or invading inferior vena cava. Portal hypertension in the mentioned case is suggested to come out of a durable cystobiliary communication and its resultant biliary cirrhosis (14,15).

## Discussion

Cystic echinococcosis or hydatid disease is mainly caused by Echinococcus granulosus. Although, this disease has been reported all over around the world, rural areas are more prone to that (1) liver is the most common affected organ. Ultrasound has emerged as a paramount diagnostic modality assessing viability of the cyst and programming treatment (2). Foreseeable complications like peritoneal rupture and biliary fistula formation make the treatment especially challenging. Fistula could be frank contributing to the evacuation of cyst contents into the biliary system or occult attributed to small tears between the cyst and biliary tree which is predominantly asymptomatic. Supposedly, the first form of fistula formation is more related to biliary complications like the entrance of daughter cysts and membrane fragments into the biliary system which may necessitate an ERCP and stent placement to reduce the risk of cholangitis and sepsis. Biliary cirrhosis has also been reported in this group of patients attributed to a longstanding cystobiliary communication (3). This scenario is consistent with our patient. Literally, the incidence of biliary fistulation is estimated 2–75% (4,5,6). Presence of cystobiliary fistula should be evaluated before surgical intervention to reduce the rate of post-operative complications like bile leakage which should be managed by external drainage. Daily drainage volumes of more than 300ml per day signals a high-flow fistula obligating an endoscopic intervention and stent placement to defer a high -risk emergent surgery to a better circumstance.(7) It was the condition we wished for. Findings have reported a relationship between cyst size ranging from 7.5-14.5 cm and biliary fistula which is comparable with cystic lesion in our patient (8,9). In addition to the cyst size, other predictive factors for cystobiliary fistula formation are seniority and presence of multiple and bilobar cysts that are associated with higher intrabiliary rupture rates. Some authors consider surgical procedures for stage 3 and 4 of cystic lesions based on Gharbi classification (10). ERCP as a diagnostic and therapeutic procedure is accepted to be used for patients with clinical, imaging and laboratory findings suspicious of frank cystobiliary fistula to decrease postoperative complication rates, and provides a shorter hospitalization time and evacuation of biliary and cystic hydatid components. ERCP with fistulotomy is the procedure of choice in patients presenting with obstructive jaundice like our patient, and is able to postpone the time of elective surgery. Failure of ERCP in this group of patients would result in an emergent surgery (7). The moot point is occult cystobiliary fistulas which are commonly seen in asymptomatic patients. Once the gradient is reversed with aspiration and evacuation of the cystic content after surgery, bile leakage begins in occult fistulas. Thus, it is believed that the evaluation of cystobiliary fistula in asymptomatic patients should be taken into consideration intraoperatively. In contrast to patients with frank fistula formation, diagnostic accuracy of ERCP and MRCP is low (7). Postoperative indications for ERCP and sphincterectomy are fistula with persistent high output, remaining hydatid element in the common bile duct or hepatic ducts and obstructive jaundice. Persistent fistulas usually close 5–7 days after sphincterectomy (11,12). Surgery has emerged as an important management strategy in these patients. Today, the laparoscopic approach replaced the conventional surgery for the treatment of liver hydatid cysts. Surgical techniques are strongly related to the location and size of hydatid cysts. Studies have revealed that laparoscopic surgery is as safe as open surgery for the majority of patients, but caution must be taken for hydatid cysts with larger size and deeper intraparenchymal location. The presence of biliary communication, as could be seen in our presented patient, can be treated with the laparoscopic technique in accordance with the open method of surgical intervention. Findings have not presented any statistically difference between the outcomes of radical cystectomy and partial pericystectomy. Therefore, partial pericystectomy is the method of choice for a large number of hydatid cysts and radical laparoscopic surgeries are withheld for only peripheral cysts (13).
